# Gonorrhœa; Its Treatment

**Published:** 1868-01

**Authors:** John M. Johnson

**Affiliations:** Atlanta, Ga.


### ARTICLE III.

*Gonorrhœa. Its Treatment.* By John M. Johnson, M. D., of Atlanta, Ga.

Finding myself so often foiled in the treatment of gonorrhœa under the various methods proposed by authors, I have for many years refused to treat cases unless the patient would consent to go to bed, and lie there until the cure is effected. Not one case in fifty is, in my opinion, cured where the subject keeps on his feet and tries to conceal his condition by attending to his business, as though nothing was the matter. Such cases may, after a while, get well, or a majority of them, but the cure is spontaneous, and owes very little to medication. Of the cases that do not recover in this way, the consequences are deplorable enough, and may continue through life.

My plan of treatment is, first to put the patient in bed, in an airy room, and make him comfortable there; then administer epsom salts and calcined magnesia, equal parts, until the bowels are completely emptied. After which, ℞—Pulv. Doveri, grs. xlviii, divide into equal portions, xii—one to be given every three or four hours until all are taken. If the ipecac in this preparation produces much nausea, then use opium or morphia in its stead. Also:

℞—Acetate Zinc., grs. xii.
Distilled Water, ℥ vi.

Mix and dissolve.

Inject half a drachm every four hours.

℞—Alum Pow'd, ʒ ii.

Toast Water, oz.

Mix and dissolve.

Let the patient drink this *ad libitum*, when drinks are called for.

The balance of the treatment consists in keeping a towel, wet with cold water, constantly to the penis, over the testicles and perineum, and on no account permit him to leave his bed or room for sixty hours.

After this, he will be weak, nervous, and sick at the stomach, but light, well-flavored broths, coffee, tea, &c., &c., will soon relieve these symptoms, and he will be as free from gonorrhœa as if he had never had it.

This treatment applies more especially to recent cases, but will succeed, with slight changes, in all the stages of gonorrhœa. Where there is persistent phlegmonous inflammation, which Ricord meets with nitrate silver, and which, according to that great author, is generally the cause of stricture, and not the powerful remedies used by him, as some assert, I rely instead upon cold water and opium—and I leave it to the candid judgment of the profession, after a careful trial, if the plan suggested above does not cure in every instance, and in one-eighth of the time required by his treatment.

In extreme cases, attended with ulceration, or even excoriation of the urethral surface, nitrate of silver is a most appropriate remedy, but I prefer the caustic bougia, because you can make your application directly to the lacuna magna, or any other part requiring it, more readily with the bougie than by injecting the fluid. I use an ointment of one grain nitrate silver to ten of fresh cerate, or lard, repeated every second day, in connection with the cold water, opium and alum water. This latter remedy has been vastly overlooked as a therapeutic agent in gonorrhœa. It expands the urethra sufficiently for practical purposes, and leaves no bad effects.

